# U-shaped kirschner wire transfixation: effective treatment for Skier’s thumb

**DOI:** 10.1186/s12893-024-02382-7

**Published:** 2024-03-15

**Authors:** Shuwei Ma, Jinzeng Zuo, Yongcheng Hu

**Affiliations:** 1 Graduate School of Tianjin Medical University, No. 22 QiXiangTai Road, Heping District, Tianjin, 300070 P. R. China; 2 Department of Orthopaedic, Tianjin Hospital, Jiefang South Road, Hexi District, Hospital, Tianjin, 300211 P. R. China; 3 The Second Hospital of Tangshan, Tangshan, 063000 Hebei P. R. China

**Keywords:** Skier’s thumb, U-shaped Kirschner wire, Ulnar collateral ligament, Metacarpophalangeal joint, Bone fragmentation

## Abstract

**Background:**

Skier’s thumb is a type of injury to the ulnar collateral ligament of the metacarpophalangeal joint of the thumb, which can result in bone fragmentation and joint instability.

**Objective:**

The objective of this study was to compare the traditional Kirschner wire fixation method with the U-shaped Kirschner wire method for treating small bone fragments with displacement, rotation, or instability in skier’s fractures.

**Method:**

A retrospective study was conducted on 30 patients with skier’s thumb who were treated at Tianjin Hospital from January 2019 to December 2021. Patients were divided into two groups: Group A received traditional Kirschner wire fixation, while Group B received U-shaped Kirschner wire fixation. Functional assessments and complications during the perioperative period were evaluated.

**Results:**

Both surgical methods significantly reduced postoperative pain and increased joint range of motion. Group B had a lower incidence of pain during follow-up and showed significant functional improvement in Tip-pinch and Grip tests compared to Group A. U-shaped Kirschner wire fixation significantly reduced complications during the perioperative period.

**Conclusion:**

The U-shaped Kirschner wire internal fixation is a safe and effective treatment for the thumb proximal phalanx base ulnar side avulsion fracture.

## Introduction

Skier’s thumb is a type of injury to the ulnar collateral ligament (UCL) of the metacarpophalangeal joint (MCPJ) of the thumb, which can result from a sudden, forceful movement or impact to the thumb [1]. The injury is characterized by the separation of a bone fragment from the main bone mass at the base of the proximal phalanx, which is the largest bone segment of the thumb. This type of injury is usually caused by the tension applied to the ligament or tendon attached to the bone during the forceful movement or impact, leading to displacement and potential instability of the joint. Treatment of Skier’s thumb depends on the severity and displacement of the fracture, which can range from immobilization to surgical intervention [2].

In a non-surgical case, Dinowitz et al. [3] reported that all of their patients experienced persistent pain. In contrast, Kuz et al. [4] and Sorene et al. [5] reported that all non-surgical treatment patients in their series were satisfied with the results, although 25% and 60% of patients did not achieve healing, respectively. In a surgical series, in the study by Boeckstyns et al. [6], the author used 6 weeks of plaster or splint fixation for fractures without involvement of the extensor pollicis brevis tendon, and the remaining cases were repaired using tendon repair. The traditional treatment for skier’s fractures is Kirschner wire fixation [7], and most studies suggest that treatment for UCL injury can achieve satisfactory results. However, there has not been a good solution for situations where bone fragmentation occurs during surgery or when the bone fragments are small and difficult to penetrate with Kirschner wire.

Non-surgical treatment of small bone fragments with displacement, rotation, and instability can easily lead to complications such as non-union, joint pain, joint instability, and traumatic arthritis. Therefore, we innovatively used the U-shaped Kirschner wire method to treat small bone fragments with displacement, rotation, or instability in skier’s fractures.

## Method

The experimental protocol was established, according to the ethical guidelines of the Helsinki Declaration and was approved by the Human Ethics Committee of Tianjin Hospital. Written informed consent was obtained from individual or guardian participant. We conducted a retrospective, continuous study of patients with skier’s thumb admitted to Tianjin Hospital from January 2019 to December 2021. The inclusion criteria were as follows: (1) ulnar avulsion fractures of the base of the proximal phalanx of the thumb; (2) positive ulnar deviation stress test; (3) fresh fractures within one week; (4) bone fragments occupying no more than 30% of the joint surface; (5) displacement of bone fragments > 1 mm or rotational deformity; and (6) closed fractures. Exclusion criteria were as follows: (1) severe osteoporosis; (2) concomitant injuries to the digital arteries, nerves, tendons, or other sites in the affected limb.

A total of 30 patients (30 thumbs) were included in this study. We compared the traditional Kirschner wire fixation method (Group A) with the U-shaped Kirschner wire method (Group B) for treating skier’s thumb. There were 15 cases in each group, with 28 male and 2 female patients; 25 right-hand and 5 left-hand injuries; ages ranging from 31 to 62 years, with an average age of 37.96 ± 12.98 years; and surgery performed within one week after injury. All surgeries were performed by the same experienced hand surgeon. General information is presented in Table 1.

**Table 1:** General Data

	Group A	Group B	t/χ^2^	*P*
No.(case)	15	15		1
Age (year)	38 ± 11	40 ± 12	0.389	0.701
Sex (Male, %)	14 (93%)	14 (93%)		1
BMI (kg/m^2^)	25.15 ± 3.23	22.93 ± 3.13	-1.904	0.067*
Follow-up (month)	13.5 ± 1.77	14.2 ± 1.47	1.122	0.271
Dominant side (right, %)	15 (100%)	13 (87%)	2.143	0.143
Occupation (case, %)	15	15	1.037	0.595
Heavy manual work	13 (87%)	14 (93%)		
Light manual work	1 (7%)	1 (7%)		
Office work	1 (7%)	0		
Time to surgery (day)	2 ± 2	4 ± 2	1.403	0.172
Operation time (min)	57 ± 13	81 ± 34	2.524	0.018*

BMI: Body Mass Index; *, *P* < 0.05

### Surgical procedure

The surgical procedure involved in this study was performed under local anesthesia with draping. Closed reduction was initially attempted by maintaining the MCPJ in 20° of flexion and interphalangeal joint in extension with the thumb in neutral adduction. The interosseous muscles were relaxed, and the thumb was placed in a position of slight ulnar deviation to ensure relaxation of the UCL. The surgeon held the edges of the proximal phalanx base on both sides and compressed them to achieve reduction. X-ray was used to confirm the alignment of the fracture line.

If reduction was not successful, or if the bone fragment was displaced outside the joint or was rotated, open reduction was performed. An arc-shaped incision was made on the ulnar dorsal side, and the skin, subcutaneous tissue, and the flexor tendon sheath were sequentially incised to expose the ulnar edge of the MCPJ, the palmar side of the base of the proximal phalanx, and the displaced bone fragment. The bone fragment was reduced and secured with a vascular clamp, and the UCL was sutured. The thumb was maintained in a relaxed position.

In Group A of patients, the traditional method was used, in which the Kirschner wire was passed through the bone fragment, penetrating both cortical bones.

**Fig. 1:**
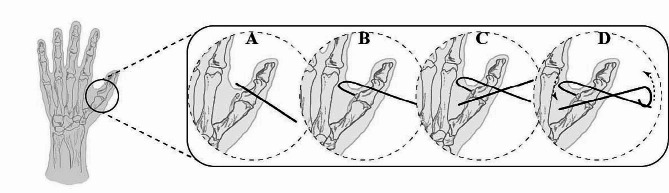
Surgical procedure. **A**: Placement of a Kirschner wire in the proximal phalanx. **B**: Bending the Kirschner wire into a “U” shape and hooking it onto the bone fragment. **C**: Insertion of the second Kirschner wire to stabilize the metacarpophalangeal joint. **D**: Bending and fixation of the two Kirschner wires at the end to form an elastic tension force, with the dashed line indicating the direction of the force

In the Group B, under C-arm guidance, the first Kirschner wire was inserted perpendicular to the fracture line, 2-3 mm distal to the fracture line of the ulnar side of the proximal phalanx and passed through the cortical bone to the radial side of the contralateral cortex (Fig. 1A). The Kirschner wire was pre-bent into a “U” shape at the insertion point, with a length of approximately 2 cm and a width of 0.5 cm. The U-shaped hook was then clamped onto the Kirschner wire and slowly withdrawn until the hook firmly engaged the bone fragment (Fig. 1B). After accurate reduction and stable fixation of the bone fragment, the second Kirschner wire was inserted into the radial side of the MCPJ towards the ulnar side of the proximal phalanx (Fig. 1C). Finally, the two Kirschner wires were interwoven and connected to form a “triangle” closed loop to maintain the elastic force of the U-shaped Kirschner wire (Fig. 1D).

After the surgical intervention, immobilization with functional position plaster was applied. X-ray imaging was conducted biweekly, starting from the fourth week, to monitor the formation of callus in the bone. The removal of Kirschner wires was determined based on specific criteria, including the presence of unclear fracture lines and the formation of continuous callus passing through the fracture line, or the disappearance of the fracture line visible on X-ray images.

### Functional assessment

**Fig. 2:**
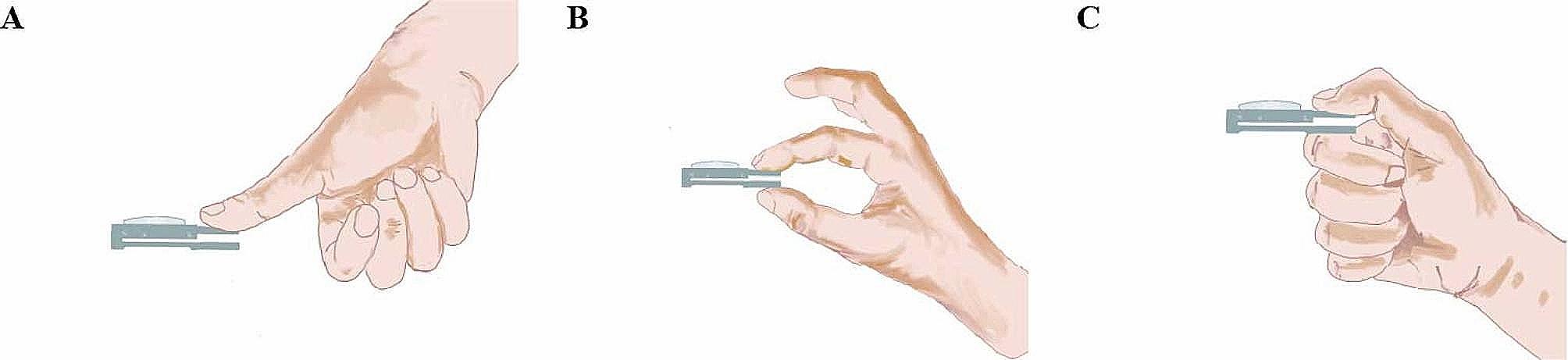
Assessment of thumb strength **A**: Opposition test; **B**: Tip-pinch test; **C**: Grip test

Complications during the perioperative period were evaluated, and the preoperative (Pre-op), postoperative (Post-op), and follow-up (Follow-up) the visual analogue scale (VAS), interphalangeal joint of the thumb (IPJ) range of motion (Fig. 2A), thumb metacarpophalangeal joint (TMCPJ) range of motion (Fig. 2B), angle of palmar abduction (Fig. 2C) were assessed at each time point.

**Fig. 3:**
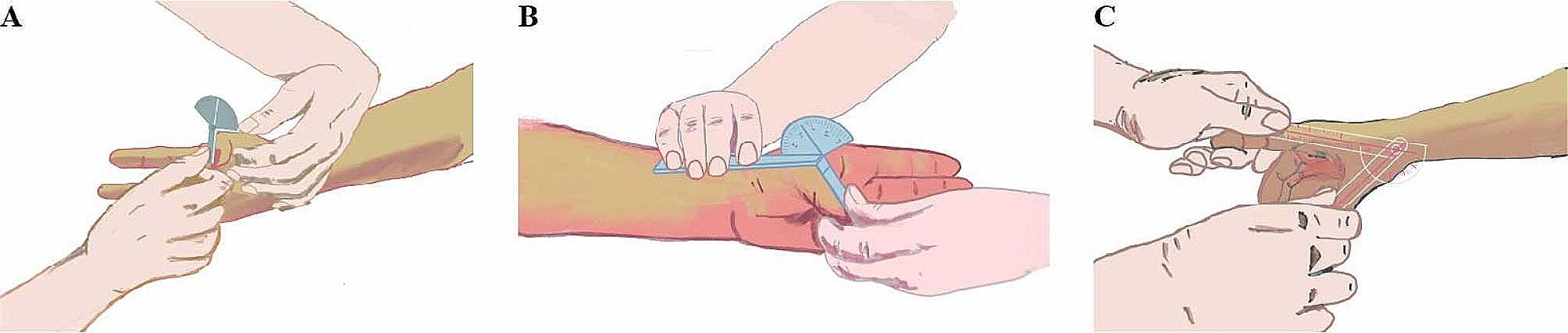
Range of Motion Test: **A**: Interphalangeal joint range of motion, **B**: Metacarpophalangeal joint range of motion, **C**: Thumb abduction angle

The hand strength of the patients was evaluated at the follow-up visit using a spring dynamometer (ZHIQU^®^, DS2-X, China) to measure the strength of opposition (Fig. 3A) tip-pinch (Fig. 3B) and grip tests (Fig. 3C) of the thumb, and the score was calculated as the affected side/healthy side * 100%.

### Statistical methods

Statistical analysis was conducted using IBM SPSS Statistics 23.0 software. Continuous variables were presented as means ± standard deviation (x ± s), and all data were approximately normally distributed or normally distributed. Paired sample t-tests were used to compare differences in preoperative, Post-op, and Follow-up measurements of the MCPJ extension angle, MCPJ range of motion, interphalangeal joint range of motion, and visual analogue scale (VAS) pain scores. Independent sample t-tests were used to compare differences in Tip-pinch, Grip, and Opposition test scores between the two groups. Chi-square tests were used to compare differences in preoperative count data and perioperative complications between the two groups. A P-value of < 0.05 was considered statistically significant.

## Results

**Fig. 4:**
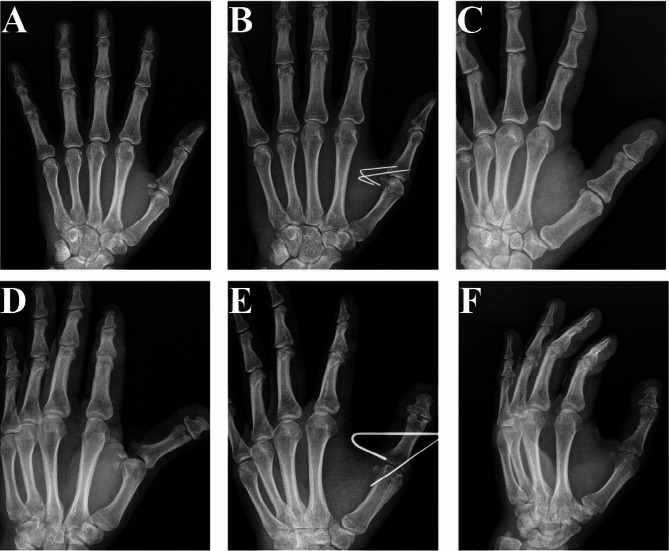
Effect images: **A**: skier’s fracture (Group A); **B**: postoperative X-ray (Group A); **C**: X-ray after removal of Kirschner wires (Group A). **D** skier’s fracture (Group B); **E**: postoperative X-ray (Group B); **F**: X-ray after removal of U-shape Kirschner wires (Group B)

All patients were followed up postoperatively for a minimum of 1 year, with an average of 13.8 ± 1.63 months. Kirschner wires were removed from 28 patients at 6 weeks postoperatively and from 2 patients at 8 weeks postoperatively, typical cases see Fig. 4. Four patients in Group A had bone fragment splitting during the operation and were fixed with sutures.

**Fig. 5:**
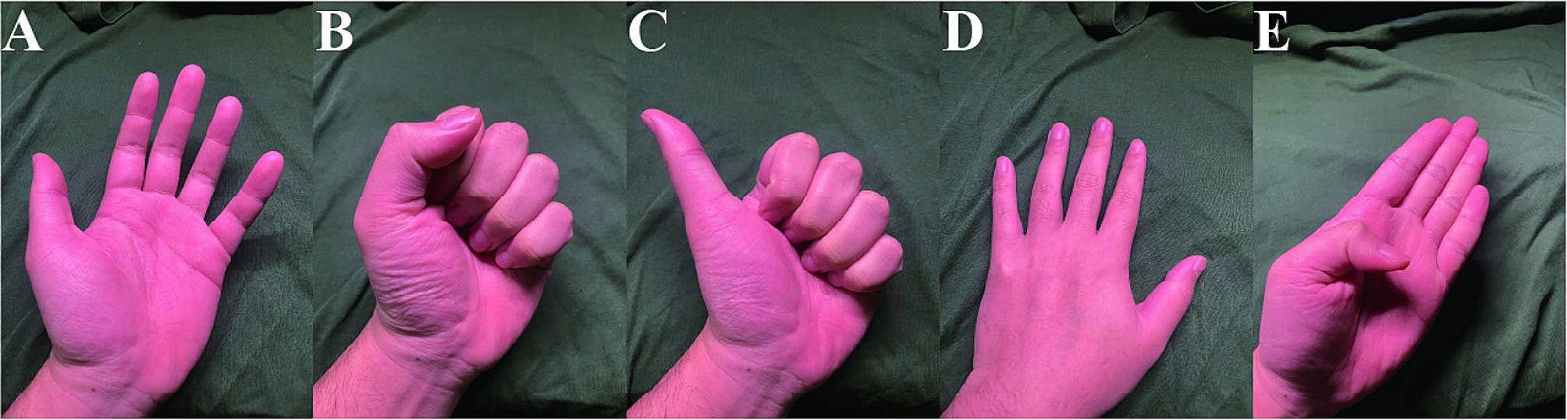
**A-E**: Postoperative 1-year follow-up functional appearance image after U-shape Kirschner wires

There were no differences in M1/M2 angle, TMCPJ motion arc, and IPJ motion arc between the two groups. However, both surgical methods significantly reduced postoperative pain compared to preoperative levels (*P*<0.05), and both methods increased M1/M2 angle, TMCPJ motion arc, and IPJ motion arc values during follow-up (*P*<0.05). Compared to Group A, Group B significantly reduced the incidence of pain during follow-up, but there was no difference in pain between the two groups postoperatively. During follow-up, there were significant differences in Tip-pinch and Grip between the two groups, with Group B showing significant functional improvement compared to Group A, but there was no difference in Opposition values between the two groups typical case see Fig. 5 (Table 2).

**Table 2:** Objective clinical outcomes

	Group A (Mean ± SD)	Group B (Mean ± SD)	*P*
Pre-op. VAS	6.20 ± 1.20	6.67 ± 1.44	0.346
Post-op. VAS	2.47 ± 1.80	2.80 ± 1.56	0.594
Follow-up VAS	2.33 ± 1.79	1.13 ± 1.06	0.036*
Pre-op M1/M2 angle (°)	39.42 ± 3.60	37.56 ± 4.94	0.248
Post-op M1/M2 angle (°)	41.61 ± 2.12	40.86 ± 1.96	0.327
follow-up M1/M2 angle (°)	55.98 ± 3.46	56.39 ± 3.24	0.742
Pre-op TMCPJ motion arc (°)	18.13 ± 2.83	18.72 ± 3.64	0.623
Post-op TMCPJ motion arc (°)	38.50 ± 3.67	36.76 ± 3.56	0.200
Follow-up TMCPJ motion arc (°)	78.92 ± 7.50	76.71 ± 9.77	0.494
Pre-op IPJ motion arc (°)	74.14 ± 8.72	75.76 ± 8.09	0.604
Post-op IPJ motion arc (°)	73.68 ± 9.46	71.48 ± 8.77	0.513
Follow-up IPJ motion arc (°)	78.93 ± 6.64	76.68 ± 10.60	0.494
Tip-pinch (%)	81.86 ± 8.15	92.20 ± 4.75	0.000*
Grip (%)	82.39 ± 11.60	91.92 ± 4.75	0.008*
Opposition (%)	87.98 ± 5.722	90.65 ± 4.91	0.181

VAS: visual analogue scale. M1/M2 angle: angle of opening of the web space between the first and second metacarpal bones; TMCPJ: thumb metacarpo-phalangeal joint; IPJ: interphalangeal joint of the thumb. *, *P* < 0.05

Secondly, U-shaped Kirschner wires were found to significantly reduce complications during the perioperative period, such as difficulty in fixing fractured bone fragments. However, there was one case of inadequate fixation with U-shaped Kirschner wire, which was treated with anesthesia and re-fixation. No patients developed osteoarthritis, thanks to the good rehabilitation treatment received by all patients, but 4 patients experienced chronic pain at 1 year, with 3 of them being the ones with bone fragment splitting during surgery (Table 3).

**Table 3:** Perioperative complications

	Group A			Group B		χ^2^	*P*
	Positive	Negative		Positive	Negative		
Bone fragmentation	4 (27%)	11 (73%)		0 (0%)	15 (100%)	4.615	0.032*
Unreliable fixation	0 (0%)	15 (100%)		1 (7%)	14 (93%)	1.034	0.309
Osteoarthritis	0 (0%)	15 (100%)		0 (0%)	15 (100%)	-	-
Nonunion of fractures	0 (0%)	15 (100%)		0 (0%)	15 (100%)	-	-
Chronic pain	4 (27%)	11 (73%)		0 (0%)	15 (100%)	4.615	0.032*

*, *P* < 0.05

## Discussion

Skier’s thumb is a rare injury, the key to treatment is to fix the bone fragments and reconstruct the UCL. The U-shaped Kirschner wire fixation method reported in this article is a method that can reduce the risk of bone fragment fragmentation caused by Kirschner wires, especially in cases where the bone fragments are small, it can achieve better results.

Most scholars believe that surgery should be the preferred treatment for fracture of the UCL of the MCPJ of the thumb [8, 9]. Moharram et al. reported that Microanchors can fix small bone fragments to anatomical reduction [10]. Shingo Komura et al. reported that Mini hook plate fixation can also fix larger bone fragments to anatomical reduction, but it was also mentioned that it is difficult to apply to smaller bone fragments [11]. S Rein et al. reported that transosseous suture fixation is a simple and effective way. Our experience suggests that it is difficult to pass the suture needle through the base bone, and the bone fragment fixation is not stable and has the risk of causing bone fragmentation. In our study, U-shaped Kirschner wire fixation can better fix small bone fragments to achieve anatomical reduction. In recent years, arthroscopic repair of UCL fractures has been proposed, which allows for derotation of the displaced avulsed bone fragment and anatomic restoration of the UCL [12, 13].

The question we are concerned about is the reliability of fixation. In the studies reported [14], Hook Plate provides stronger fixation for thumb UCL avulsion fracture than Suture Anchor Fixation for fixable bone fragments. Most studies do not report the risk of bone fragmentation caused by bone puncture devices (screws, Kirschner wires, anchor pins). This is also a practical problem we have encountered in clinical practice. Our study focuses on the first surgical treatment after acute injury, and we also apply U-shaped Kirschner wire fixation to fix bone fragments that have been fragmented due to bone puncture devices.

The thumb accounts for 40% of hand function [15]. Due to pain caused by UCL fractures, clinical examination shows tenderness at the base of the thumb, and UCL injuries can affect pinching or gripping activities such as grasping an object, turning a key or doorknob, and writing. Therefore, postoperative evaluation of these functions is the best feedback on surgical effectiveness. Katolik, Leonid I et al. found that Bone Anchor can achieve a range of motion of 97% of the healthy side, but pull-out suture can only reach 86% [16]. In our study, although we cannot reach the 97% reported, we have achieved 92% effectiveness. Conservative treatment is obviously not a good method. A study reported that 15% of patients demonstrated persistent instability and pain at 12 weeks and were treated with surgical reconstruction.

The limitation of our study is that preoperative magnetic resonance imaging was not thoroughly conducted, and only physical examination and ultrasound were used to determine the rupture of the UCL. In previous studies, there was an approximately 3% misdiagnosis rate [2]. In addition, the small sample size in our study may cause certain bias.

In conclusion, the U-shaped Kirschner wire internal fixation is a safe and effective treatment for the thumb proximal phalanx base ulnar side avulsion fracture. Compared with traditional treatment methods, the U-shaped Kirschner wire technique is simpler, more cost-effective, and has lower complication risks. Although this method has some limitations, especially in patients with large bone fragments or severe displacement, it provides some advantages, including greater stability and the ability to achieve bone healing without removing small bone fragments. Future research needs to further evaluate the long-term results and complications of U-shaped Kirschner wire fixation and compare it with other treatment options in a larger patient population.

## Data Availability

The datasets used or analyzed during the current study are available from the corresponding author on reasonable request.
